# Aberrant caspase-activated DNase (CAD) transcripts in human hepatoma cells

**DOI:** 10.1038/sj.bjc.6600695

**Published:** 2003-01-28

**Authors:** S Y Hsieh, S F Liaw, S N Lee, P S Hsieh, K H Lin, C M Chu, Y F Liaw

**Affiliations:** Liver Research Unit, Chang Gung Memorial Hospital, 199 Tung-Hua North Road, Taipei, Taiwan; Institute for Basic Medical Research, Chang Gung University, Taoyuan; Department of Chemistry, Fu-Jen University, Hsin-Chuang, Taipei, Taiwan

**Keywords:** caspase-activated DNase, DNA fragmentation factor, DFF, DFF-B, apoptosis, hepatocellular carcinoma

## Abstract

The gene of caspase-activated DNase (CAD), the key enzyme for nucleosome cleavage during apoptosis, is mapped at chromosome 1p36, a region usually associated with hemizygous deletions in human cancers, particularly in hepatoma (HCC). It is tempting to speculate that CAD plays a tumour-suppressive role in hepatocarcinogenesis. To address this, we examined the CAD transcripts in six human HCC cell lines, one liver tissue from a non-HCC subject, and peripheral blood leukocytes (PBL) from three healthy individuals. Alternatively spliced CAD transcripts with fusion of exon 1 to exon 7 were isolated in most of the examined samples including HCC cells and normal controls. However, relatively abundant alternatively spliced CAD transcripts with fusion of exon 2 to exon 6 or 7, in which the corresponding domain directing CAD interaction with ICAD was preserved, were found only in poorly differentiated Mahlavu and SK-Hep1 cells. Interestingly, an abnormal CAD transcript with its exon 3 replaced by a truncated transposable *Alu* repeat was isolated in Hep3B cells, indicative of the implication of an *Alu*-mediated genomic mutation. Moreover, mis-sense mutations in the *CAD* genes were identified in all six HCC cell lines. Upon UV-induced apoptosis, DNA fragmentation efficiency was found to be intact, partially reduced and remarkably reduced in Huh7 and J328, Hep3B and HepG2, and Mahlavu cells, respectively. That mutations and aberrantly spliced transcripts for the *CAD* gene are frequently present in human HCC cells, especially in poorly differentiated HCC cells, suggests a significant role of CAD in human hepatocarcinogenesis.

Apoptosis is a programmed cell death process that removes toxic or useless cells during mammalian development (Wyllie, 1980). The biological hallmark of apoptosis is the cleavage of chromosomal DNA into nucleosomal fragments (Wyllie, 1980). Human caspase-activated DNase (CAD, also called DFF40/CPAN) is the key enzyme for nucleosome cleavage during cell apoptosis (Liu *et al*, 1997; Enari *et al*., 1998; Halenbeck *et al*, 1998; Mukae *et al*, 1998). CAD contains two different functional domains: the N-terminal regulatory domain (CIDE-N or CAD domain) and the C-terminal catalytic domain. The CAD domain shares sequence homology with the N-terminal of the inhibitor of CAD (ICAD) (Liu *et al*, 1997; Mukae *et al*., 1998). Structural and mutational analyses of the CAD domains revealed that they account for the ability of CAD and ICAD to form a heterodimer (Inohara *et al*, 1999; Lugovskoy *et al*, 1999; Uegaki *et al*, 2000; Zhou *et al*, 2001). ICAD inhibits CAD activity by binding to CAD. During the apoptotic process, CAD is released from the ICAD/CAD complex via proteocleavage of the ICAD by caspase 3 or caspase 7. CAD then degrades chromosomal DNA. Moreover, ICAD also works as a chaperon to help the correct folding of the CAD protein (Enari *et al*, 1998; Zhang *et al*, 1998; Sakahira *et al*., 1999). The active CAD can be synthesised only in the presence of ICAD both *in vitro* and *in vivo*. The CAD synthesised *in vitro* without ICAD does not have DNase activity at all.

Inactivation of CAD has been shown to render cells resistant to undergoing apoptosis (Sakahira *et al*, 1998) and may cause cell transformation. Recently, human *CAD* gene was mapped at chromosome 1p36.3 (Mukae *et al*, 1998). Human chromosome 1p36 is frequently associated with hemizygous deletions in several types of cancers, particularly in hepatocellular carcinoma (HCC), indicating the presence of one or more tumour suppressor genes in the region (Buetow *et al*, 1989; Simon *et al*, 1991; Yeh *et al*, 1994). It is, therefore, tempting to speculate that CAD might be one of the tumour suppressors for hepatocellular carcinogenesis. To address this hypothesis, we examined the CAD transcripts in human HCC cells.

## MATERIALS AND METHODS

### Hepatoma cell lines, liver tissue and peripheral leukocytes

Human hepatoma cell lines, HepG2, Hep3B, Huh7, Sk-Hep1 (Fogh *et al*, 1977; Chang *et al*, 1993), and Mahlavu were obtained from American Type Culture Collection (Manassas, VA, USA), while J328 was kindly provided by Dr CS Yang (Chen *et al*, 1994). All media were supplemented with 10% (v/v) foetal bovine serum and 2 mM L-glutamine. One non-HCC liver sample, obtained from a patient who received liver resection on account of a benign focal nodular hyperplasia of the liver, and peripheral blood leukocyte (PBL) samples obtained from three healthy individuals were included in the study as non-HCC controls. The liver sample was frozen immediately in liquid nitrogen after resection and stored at −80°C until processing. The Ethics and Science Committee of Chang Gung Memorial Hospital approved specimen collection procedures and informed consent was obtained from each subject or subject's guardian.

### RNA preparation and RT–PCR

**Figure 1:**
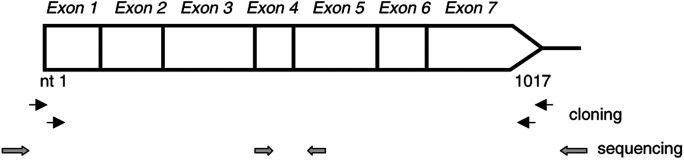
Schematic representation of the strategies for cloning and sequencing of the CAD cDNA. The CAD reading frame was cloned using nested RT–PCR method with two rounds of PCR following reverse transcription. Two primer pairs for amplification of the nested PCR are shown (arrows in both ends of the CAD reading frame). After examination with gel electrophoresis, the PCR products were cloned directly and then subjected to sequencing analysis using two vector primers (stippled arrows) flanking the cloning site and two CAD-specific primers (stippled arrows inside the reading frame).

Total cellular RNA was extracted from HCC cell lines, liver tissue and peripheral blood leukocytes using the single-step acid–phenol extraction method as described before (Lin *et al*, 2001). Total RNA (2 *μ*g) was converted to cDNA via a random-primer method as described previously (Hsieh *et al*, 1997). The whole reading-frame of the *CAD* gene was cloned using nested PCR (Figure 1A

). In brief, 1/50th of the cDNA was subjected to amplification for the first round of PCR in a total volume of 50 *μ*l, which contained 4 pmol of each primer, 200 nM of each dNTP, and 2 U of Taq DNA polymerase in a buffer containing 40 mM Tricine-KOH (pH 8.7), 3.5 mM MgCl_2_, 15 mM KOAc, 0.005% Tween-20 and 0.005% Nonidet-P40 (Advantage 2 PCR kit, CLONTECH, Palo Alto, CA, USA). Amplification was performed using 30 cycles of 95°C for 30 s, 65°C for 4 min. The first-round PCR products were diluted 1/50 and 1 *μ*l of the dilute was subjected to the second round of PCR with the inner primer pairs using conditions similar to that of the first-round PCR. After gel-electrophoresis analysis, the second-round PCR products were directly cloned using a PCR-TA cloning kit (pCRII TOPO, Invitrogen, Carlsbad, CA, USA). At least 10 clones derived from each HCC cell line and the liver sample, and at least five clones from each peripheral blood leukocyte samples were randomly selected for sequencing analysis. Sequencing analysis, using the two primers encoded in the vector and the two CAD-specific primers as illustrated in Figure 1a, was conducted as described before (Hsieh *et al*, 1999).

The primer sequences for the nested PCR were as follows:

First-round PCR: 5′-cccagagggcttgaggacatctgcaa-3′
5′-ACCAGGACGTGGTGTGTACGTGTCA-3′
Second-round PCR: 5′-gaggacatctgcaatgctccagaagc-3′
5′-TCACTGGCGTTTCCGCACAGGCTG-3′

The sequences of the primers for sequencing are:

T7 primer: 5′-gtaatacgactcgctataggg-3′
SP6 primer: 5′-tatttaggtgacactatag-3′
CAD sense: 5′-GTGGTTTGAACGCTTGCAGTCCCCGA-3′
CAD antisense: 5′-gaccggagcctctggcacgtgga-3′

The nucleotide numbering used in this report is according to the numbering system by Mukae *et al* (1998) (GenBank accession numer: AB013918).

### UV irradiation and DNA fragmentation

At 24 h after plating (2×10^6^ /100 mmdish), cells were irradiated with 50–200 mJ cm^−2^ of UV and harvested for DNA fragmentation at 60, 90, 120, 150, 180 and 210 min thereafter, respectively. PBS-washed cells were suspended in DNA extraction buffer containing 50 mM Tris (pH 8.0), 10 mM EDTA, 0.5% *N*-laurosarcosine and 1 mg ml^−1^ proteinase K, and incubated for 1 h at 50°C in a water bath. Then DNase-free RNase was added at a final concentration of 250 *μ*gml^−1^, at 50°C for another hour followed by ethanol precipitation of DNA. The DNA pellets were resuspended in TE buffer (10 mM Tris, pH 8.0/1 mM EDTA). DNA (1 *μ*g) was end labelled with *α*^32^P-dATP in a solution containing 1 *μ*g DNA, 0.5 mCi *α*^32^P-dATP, 5 U Klenow polymerase, 10 mM Tris, pH 7.5, 5 mM MgCl_2_ for 30 min at room temperature. The reaction was terminated by adding an equal volume of 10 mM EDTA. The labelled DNA was purified via a Sephadex G-50 spin-column (Pharmacia Biotech, Quarry Bay, Hong Kong), and analysed by electrophoresis on a 1% agarose gel and then by autoradiography. For quantification of DNA fragmentation upon UV-induced apoptosis in each HCC cell line, we used a cellular DNA fragmentation ELISA kit in accordance with the manufacturer's instruction (Roche Diagnostics GmbH, Mannheim, Germany). In brief, cells were metabolically labelled with 10 *μ*M of thymidine analogue BrdU for overnight. At 4 h after UV irradiation with 0, 100 or 200 mJ cm^−2^, cell membrane was lysed for 30 min at 20°C in a solution containing 10 mM EDTA and 0.1% Tween-20. Following centrifugation for 10 min at 2500 ***g*** to remove cellular debris and nucleus component, the cytoplasmic lysate was subjected to an ELISA to detect fragmented DNA in cytoplasm.

## Results

**Figure 2:**
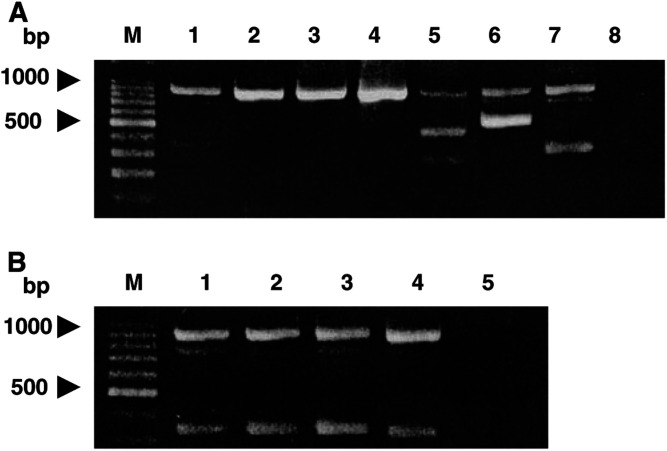
Expression of the *CAD* gene by nested RT-PCR. After reverse transcription, the samples derived from six HCC cell lines were subjected to two rounds of PCR as described in the legend of Figure 1. (**A**) Lanes 1–8 represent the size marker, the PCR products derived from HepG2, Hep3B, Huh7, J328, Mahlavu, SK-Hep1 cells and the non-HCC liver sample, respectively. Of note, HepG2 and Hep3B are well-differentiated HCC cells, Huh7 and J328 are moderately differentiated HCC cells, and Mahlavu and SK-Hep1 are poorly differentiated HCC cells. (**B**) Lanes 1–5 represent the PCR products derived from the non-HCC liver sample and from the three PBL samples and a negative control for PCR respectively.

To study abnormalities in the CAD transcripts in HCC, we reverse transcribed mRNA and amplified the complete CAD coding sequences by nested PCR (Figure 1). As illustrated in Figure 2

, the 1-kb PCR products, which represented the full-length CAD transcripts, were detected in all the examined samples. Small PCR products, about 0.34 kb, representing sub-full-length CAD transcripts were found in the non-HCC liver sample and in PBL from the three normal individuals (Figure 2B), whereas 0.45-kb and 0.55-kb PCR products for sub-full-length CAD transcripts were detected in Mahlavu and SK-Hep1 cells, respectively (Figure 2A, lanes 5 and 6). The PCR products were directly cloned. At least 10 clones derived from each hepatoma cell line and non-HCC liver sample and at least five clones derived from each PBL sample were randomly selected for sequencing analysis. The sequencing results have been deposited in the GenBank and are summarised in Table 1

**Table 1:** Variants of CAD cDNA in human HCC cell lines, non-HCC liver tissue and PBL

		**The CAD cDNA clones**							
**Cell line**	**Exon 1**	**2**	**3**	**4**	**5**	**6**	**7**		
HepG2	▪	▪	▪	▪	▪	▪	▪	Prototype
	*	▪	▪	▪	*	▪	▪	G33S/S225
	▪	▪	∣			∣	▪	▵367–702
								
Hep3B	▪	▪	▪	▪	*	▪	▪	R196K
	▪	▪	A	▪	▪	▪	▪	Alu repeat replacement
								
Huh7	▪	▪	▪	▪	▪	▪	▪	Prototype
	▪	*	▪	▪	▪	▪	▪	L89P
	▪						▪	Exons 1–7
								
J328	▪	▪	▪	▪	▪	▪	▪	SNP ACG177ACA
	▪	▪	*	*	▪	▪	▪	E110Q/Q150R
	▪						▪	Exons 1–7
								
Mahlavu	▪	▪	▪	▪	*	▪	▪	V171A
	▪	▪					▪	Exons 1–2–7
								
SK-Hep1	▪	▪	▪	▪	*	▪	*	E184L/K264T
	▪	▪				▪	▪	Exons 1–6–7
								
Non-HCC liver	▪	▪	▪	▪	▪	▪	▪	Prototype
	▪						▪	Exons 1–7
								
PBL	▪	▪	▪	▪	▪	▪	▪	Prototype
	▪	▪		▪	▪	▪	▪	Skipping exon 3
	▪						▪	Exons 1–3
								
PBL	▪	▪	▪	▪	▪	▪	▪	Prototype
	▪	▪		▪	▪	▪	▪	Skipping exon 3
	▪						▪	Exons 1–7
								
PBL	▪	▪	▪	▪	▪	▪	▪	Prototype
	▪						▪	Exons 1–7

* mis-sense mutation; A: the Alu repeat; ▵: deletion with CTGC repeats at both ends; ∣: incomplete exon sequences.

.

**Figure 3:**
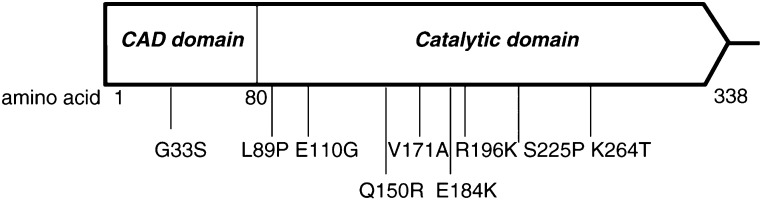
Scheme of mapping the mis-sense mutations to the corresponding CAD reading frame. Amino acids 1–80 is the CIDE-N or CAD domain that directs interaction with ICAD; the C-terminal is the catalytic domain containing nuclease activity.

The prototype transcripts were found in HepG2, Huh7 cells, non-HCC liver sample and the three PBL samples (Table 1). The full-length transcripts with single-nucleotide polymorphism (SNP) without change of amino acid were identified in J328 cells (wild type, in terms of amino-acid sequences). In addition, the full-length transcripts with SNP leading to amino-acid changes (mis-sense mutations) were noted in all of the 6 HCC cell lines (Table 1). All these mis-sense mutations corresponding to the CAD reading-frame are illustrated in Figure 3

. No hotspot for these mis-sense mutations in CAD was found, although most of them were located in the catalytic domain of CAD.

**Figure 4:**
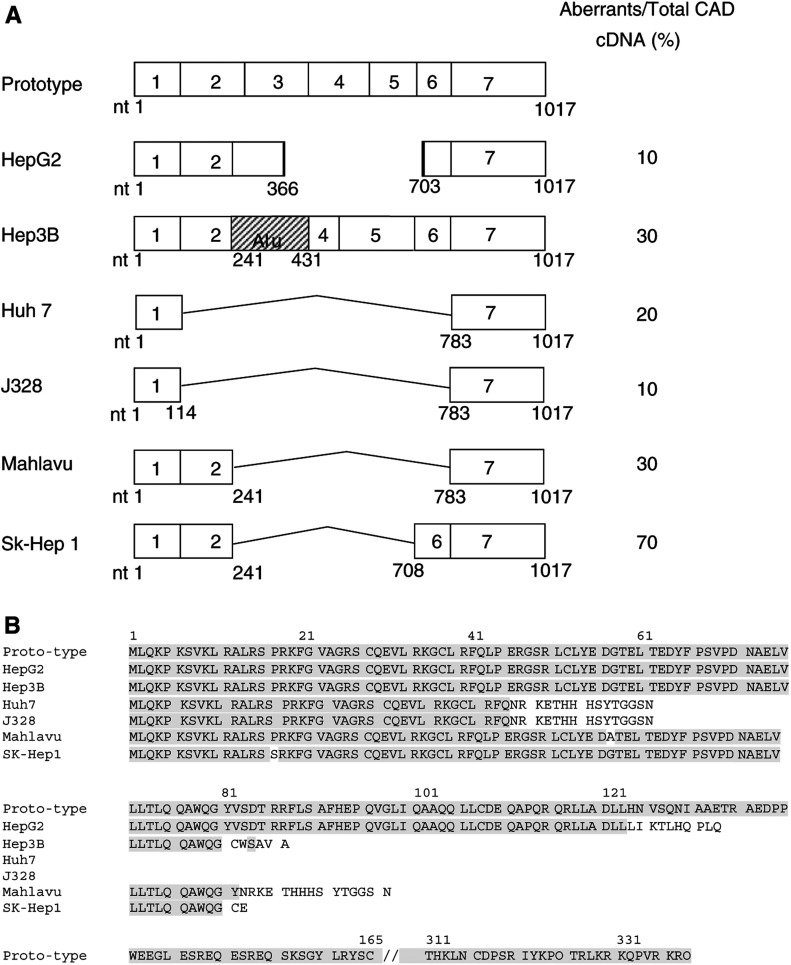
Scheme of the aberrant CAD transcripts and their predicted proteins. (**A**) The coding exons 1–7 are demonstrated as open squares. The heavy lines in the transcripts in HepG2 cells represent a four-nucleotide identity at the two skipping ends (nucleotides 367–370 and 702–705, in accordance with the numbering system by Mukae *et al* (1998))). The box labelled with ‘Alu’ in Hep3B cells represents a sequence homologous to a human *Alu* repeat. (**B**) Alignment of the predicted amino-acid sequences for the aberrant CAD transcripts to the prototype CAD amino-acid sequences (Mukae *et al*, 1998). Note that CAD domain (aa 1–80) is preserved in the aberrant transcripts from HepG2, Hep3B, Mahlavu and SK-Hep1 cells.

Sub-full-length transcripts were found in all of the six hepatoma cell lines (Figure 4A

), even though sub-full-length transcripts were detected by gel electrophoresis only in Mahlavu and SK-Hep1 among the six examined HCC cell lines. In HepG2 cells, a minor sub-full-length transcript (one out of the 10 selected clones) with a deletion from nt 367 to nt 702 was identified. Of note, a four-nucleotide identity, CTGC, was noted exactly at both of the 5′- and 3′-deletion ends (nucleotides 367–370 and 703–706), suggesting involvement of a recombination deletion. In Hep3B cells, an aberrant CAD transcript (three out of the 10 clones) corresponded to replacement of exon 3 with a 121-base sequence, which was homologous to part of the human transposon, *Alu* repeat. In both Huh7 and J328 cells, there were minor small transcripts (two and one out of the 10 clones, respectively) with the absence of exons 2–6 resulting in conjunction of exon 1 to exon 7, suggestive of transcripts derived from an alternative splicing process. Similar alternatively spliced CAD transcripts with fusion of exon 1 to exon 7 were also noted in the non-HCC liver tissue and the three PBL samples as well (Figure 2B and Table 1). In Mahlavu and SK-Hep1 cells, relatively abundant alternatively spliced transcripts (three and seven out of the 10 clones, respectively) coincided with the absence of exons 3 to 6, or to 5 leading to fusion of exon 2 to exon 7 or 6, respectively (Table 1 and Figure 4A).

The predicted amino-acid sequences for these aberrant transcripts are aligned to that of the prototype CAD and shown in Figure 4B. Of note, the amino-acid sequences for CAD domain (aa 1–80) were preserved in the aberrant CAD transcripts identified in HepG2, Hep3B, Mahlavu and SK-Hep1 cells.

**Figure 5:**
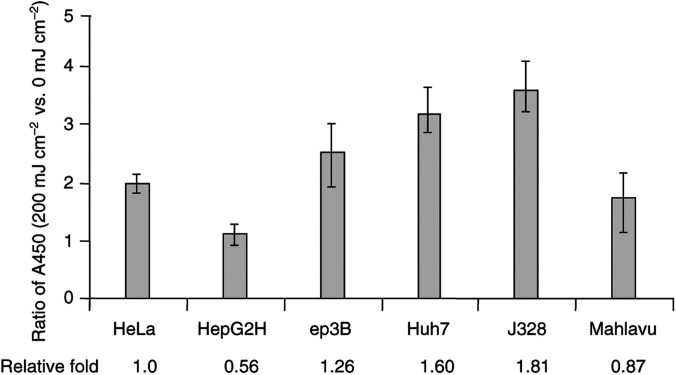
ELISA for cellular DNA fragmentation. After 0, 100 or 200 mJ cm^−2^ of UV irradiation, cytoplasmic lysate of cells containing DNA metabolically prelabelled with BrdU was subjected to an ELISA with anti-DNA antibody binding to fragmented DNA in cytoplasm followed by quantification with a peroxidase-conjugated anti-BrdU antibody (Roche Diagnostics GmbH, Mannheim, Germany). The representative results were derived from experiments conducted in a manner of duplication for each sample in every assay in a total of three assays. The averages and ranges of the ratio of DNA fragmentation before *vs* after UV irradiation for each cell line are illustrated. The relative amount of DNA fragmentation for each HCC cell line was determined via comparison to that for HeLa cells.

To inspect the nucleosome cleavage activity of CAD during apoptosis in these HCC cell lines, DNA fragmentation assay was conducted after UV induction of apoptosis. As shown in Figure 5

, the relative efficiency of DNA fragmentation upon UV irradiation in HepG2, Hep3B, Huh7, J328 and Mahlavu cells was 0.56-, 1.26-, 1.60-, 1.81- and 0.87-fold, respectively, as compared to that in HeLa cells. The relative inefficiency of DNA fragmentation upon UV irradiation in HepG2 cells was attributable to a relatively low susceptibility to UV-induced apoptosis (data not shown).

## DISCUSSION

Herein we report the identity of aberrant CAD transcripts in human HCC cells. Two classes of human CAD cDNA have been cloned by Mukae *et al* (1998). One encompasses the entire coding region, whereas the other contains many deletions, insertions and point mutations in the corresponding coding region. The latter is thought to be derived from a pseudogene (Mukae *et al*, 1998). Obviously, the subfull-length CAD cDNAs reported herein were not derived from pseudogenes. Instead, most of them were more likely generated via aberrant RNA processing mechanisms.

The minor CAD transcript (representing one out of the 10 isolated CAD cDNA clones) in HepG2 cells with a deletion from nucleotides 367 to 702 possibly resulted from a recombination processing, because of a four-nucleotide identity, CTGC, noted exactly in both the deletion ends (nucleotides 367–370 and 702–705, respectively). Two different species of full-length CAD transcripts, a prototype and a transcript with mis-sense mutations, were identified, suggesting that the recombination occurs more likely during mRNA processing than at the chromosomal DNA level (Table 1).

The sub-full-length CAD transcript (representing three out of the 10 isolated CAD cDNA clones) corresponding to replacement of exon 3 with a truncated human transposon, *Alu* repeat, was isolated in Hep3B cells. *Alu* elements are a specific human family of interspersed repetitive sequences. It was estimated to be over 1000 000 copies per genome. Their dispersion and plethora is attributable to transposition via a mechanism that has not been fully understood yet (Mathias *et al*, 1991). It was likely that the CAD transcript with replacement of exon 3 with the *Alu* element reported herein was generated via an *Alu*-mediated genomic mutation mechanism. One possible mechanism is that a *de novo* *Alu*-insertion mutation occurs in one of the two alleles of the *CAD* gene. In theory, mobilization of *Alu* elements requires a cellular source of reverse transcriptase that is generally believed to be provided by a concurrently activated retrotransposon, such as *LINE-1* elements (Mathias *et al*, 1991). Indeed, global DNA demethylation has been generally found in human cancers including HCC (Lin *et al*, 2001), which in turn leads to activation of transposable elements, such as *LINE-1* and *Alu* repeats. It is, therefore, conceivable to speculate that mutations secondary to transposition of transposons in human cancers may not be rare events. Indeed, *de novo Alu* insertion leading to human diseases, particularly in tumours, has been reported before (Kazazian, 1998). For examples, the insertions cause heamophilia B (factor *IX* gene) (Vidaud *et al*, 1993), neurofibromatosis (*NF-1* gene) (Walllace *et al*, 1991), Apert syndrome (*FGFR2* gene), acholinesterasemia (*ChE* gene) (Muratani *et al*, 1991), desmoid tumors (*APC* gene) and breast cancer (Katagiri and Nakamura, 1996) (see the review by Kazazian, 1998). Alternatively, this *Alu*-containing CAD transcript might result from an *Alu*/*Alu* homologous recombination/deletion, which led to exon 3 deletion and activation of a cryptic splicing acceptor site in *Alu* elements. Experiments to elucidate the roles of *Alu*-elements in genomic mutations for the *CAD* gene are in progress.

On the other hand, the sub-full-length CAD transcripts found in Huh7 and J328 cells exhibited loss of exons 2–6 and resulted in fusion of exons 1 and 7. Since the fusion junctions just coincided with the splice sites, they must be generated via alternative splicing processes. Similar alternative spliced transcripts with fusion of exons 1–7 were also found in the liver tissue from a non-HCC subject and in the PBL from three healthy individuals. The CAD reading frame encoded by these small transcripts was so greatly disrupted that they might not have any biological significance.

By contrast, the sub-full-length CAD transcripts found in poorly differentiated HCC cells, Mahlavu and SK-Hep1, corresponded to the absence of exons 3 to 6 or 5 and created a junction between exon 2 to exon 7 or 6, respectively. Apparently, they were generated via aberrant splicing mechanisms. These transcripts are of great interest because not only were they cancer specific (only found in poorly differentiated Mahlavu and SK-Hep1 cells) and relatively abundant (30 and 70% of the isolated CAD cDNA clones, respectively), but they also preserved the CAD domain (aa 1–80) directing CAD interaction with its inhibitor (ICAD) (Mukae *et al*, 1998; Uegaki *et al*, 2000, Otomo *et al*, 2000). Since ICAD functions as a chaperon for the regulation of CAD activity (Enari *et al*, 1998; Sakahira *et al*, 1998; Sakahira *et al*, 2000), these predicted aberrant CAD products might sequester ICAD from interacting with the wild-type CAD, thereby preventing the correct folding of CAD and subsequently blocking the nuclease activity of the wild-type CAD in the cells. Interference of the CAD activity renders cells more resistant to cell death, and in turn may award cells with advantages for survival and, possibly, for carcinogenesis (Sakahira *et al*, 1998). It is, therefore, intriguing to speculate that these aberrant CAD products encoded by the aberrant CAD transcripts in SK-Hep1 and Mahlavu cells (and those in HepG2 and Hep3B cells, Figure 4A and B) play significant roles in cell apoptosis and in hepatocarcinogenesis. Indeed, in the experiments for DNA laddering under UV-induced apoptosis, the phenomenon of DNA laddering was remarkably impaired in Mahlavu and SK-Hep1 cells, and partially impaired in HepG2 and Hep3B cells, as compared to that in HeLa, Huh7 and J328 cells. Studies to further dissect the effects of these C′- truncated CAD on nucleosome cleavage activities of the wild-type CAD are currently ongoing.

Alternative splicing has been regarded as an important mechanism that contributes to genetic diversity by generating multiple protein isomers from a single gene (Croft *et al*, 2000; Mironov *et al*, 2000). However, alternative splicing can also lead to human disease (Blencowe, 2000; Philips and Cooper, 2000). Alterations in alternative splicing of certain pre-mRNA have recently been correlated to either neoplastic transformation and/or acquisition of metastatic potential (Savagner *et al*, 1994; Cooper and Dougherty, 1995; Barrack, 1996; Pfeffer, 1996; Skuse and Cappione, 1997; Iwase *et al*, 1998; Philips and Cooper, 2000). Most of these altered splicing patterns are because of either changes in *trans*-acting factors or mutations within the canonical sequences at the intro/exon border that are required for splicing. However, mutations in an auxiliary element required for pre-mRNA splicing within exons were found more recently. It was reported that in human neuroblastomas, ataxia telangiectasia and breast cancer some nonsense mutations caused skipping of exons, a phenomenon known as nonsense-associated altered splicing (Teraoka *et al*, 1999; Ars *et al*, 2000; Hastings and Krainer, 2001; Liu *et al*, 2001). The latter was attributable to result from non-sense mutations in exon leading to disrupting a splicing enhancer in exon, which contains recognition sequences for splicing factors, such as SF2/ASF (Coulter *et al*, 1997; Hastings and Krainer, 2001; Liu *et al*, 2001). The exon-skipping phenotype has been reproduced *in vitro* and was shown not to result from disruption of the translational reading frame (Blencowe, 2000). The canonical sequences at the intron-2/exon-3 junction and the coding sequences in exon 3 for the CAD genes in Hep3B, Mahlavu and SK-Hep1 cells have been examined. However, neither mutations to disrupt the intro/exon sequence nor non-sense mutations in exon 3 was found (data not shown). Further studies to elucidate the underlying molecular mechanism for altered alternative splicing for the *CAD* gene in HCC are warranted.

In summary, we report the identity of aberrant transcripts and frequent mutations for the *CAD* gene in human HCC cells, suggesting a significant role of CAD in human hepatocarcino-genesis.
